# Supplementary material for: Identification of photoperiod-induced specific miRNAs in the adrenal glands of Sunite sheep (Ovis aries)
Source: Front Vet Sci. 2022 Jul 22;9:888207. doi: 10.3389/fvets.2022.888207 (PMC9354845; doi:10.3389/fvets.2022.888207)
Supplement: Supplementary file 1 [file Data_Sheet_1.ZIP › Supplementary file/miRDeep2 file.zip!/miRDeep2 file/Know_Structure/LP42A3/oar-mir-29b-1.pdf]

miRBase precursor : oar-mir-29b-1
Total read count : 14564
oar-miR-29b read count : 13692
remaining reads : 872

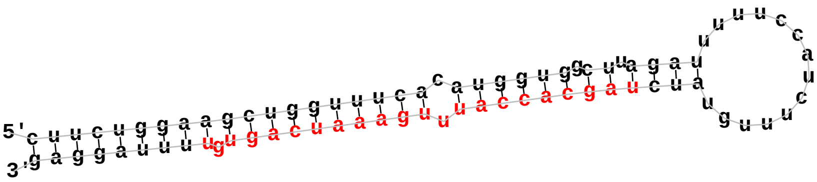

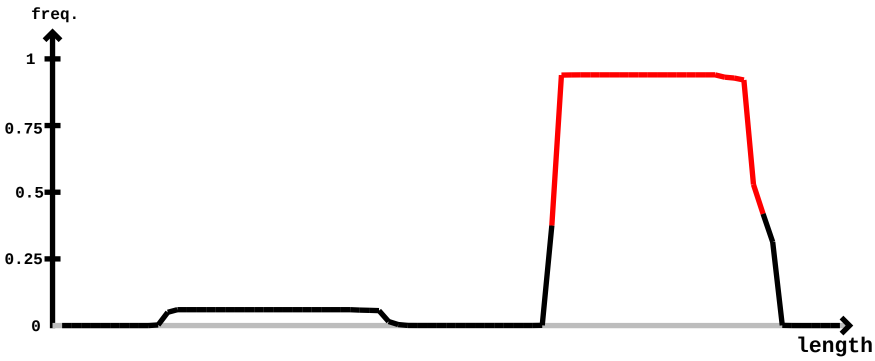

oar-miR-29b

```
5'- cuucuggaagcugguuucacaugguggcuuagauuuuuccaucuuuguaucuagcaccauuugaaaucaguguuuuaggag   -3'    exp
    ((((((((((((((((((.((((((.((.((((...............))))))))))))).)))))))))).)))))))))    reads     mm        sample
    .........gcugguuucacaugguggcuu...................................................    5         0         seq
    .........gcugguuucacaugguggcuua..................................................    3         0         seq
    .........gcugguuucacaugguggcuuag.................................................    1         0         seq
    .........gcugguuucacaugguggcuuaga................................................    25        0         seq
    .........gcugguuucacaugguggcuuagau...............................................    1         0         seq
    ..........cugguuucacaugguggcuu...................................................    15        0         seq
    ..........cugguuucacaugguggcuua..................................................    11        0         seq
    ..........cugguuucacaugguggcuuag.................................................    8         0         seq
    ..........cugguuucacaugguggcuuaga................................................    503       0         seq
    ..........cugguuucacaugguggcuuagau...............................................    127       0         seq
    ..........cugguuucacaugguggcuuagauu..............................................    25        0         seq
    ..........cugguuucacaugguggcuuagauuu.............................................    1         0         seq
    ...........ugguuucacaugguggcuu...................................................    2         0         seq
    ...........ugguuucacaugguggcuuag.................................................    7         0         seq
    ...........ugguuucacaugguggcuuaga................................................    70        0         seq
    ...........ugguuucacaugguggcuuagau...............................................    38        0         seq
    ...........ugguuucacaugguggcuuagauu..............................................    23        0         seq
    ...........ugguuucacaugguggcuuagauuu.............................................    2         0         seq
    .................................uuuuuccaucuuuguauc..............................    5         0         seq
    .................................................ucuagcaccauuugaaaucagu..........    8         0         seq
    ..................................................cuagcaccauuugaaau..............    1         0         seq
    ..................................................cuagcaccauuugaaauc.............    50        0         seq
    ..................................................cuaCcaccauuugaaauc.............    1         1         seq
    ..................................................cuagcaccauuugaaauca............    12        0         seq
    ..................................................cuagcaccauuugaaaucag...........    18        0         seq
    ..................................................cuagcaccauuugaaaucagu..........    3234      0         seq
    ..................................................cuagcaccauuugaaaucGgu..........    26        1         seq
    ..................................................cuagcaccauuugaaaucagug.........    1151      0         seq
    ..................................................cuagcaccauuugaaaucagugu........    948       0         seq
    ..................................................cuagcaccauuugaaaucaguguu.......    10        0         seq
    ...................................................uagcaccauuugaaauU.............    6         1         seq
    ...................................................uagcaccauuugaaauc.............    67        0         seq
    ...................................................uagcaccauuugaaauA.............    2         1         seq
    ...................................................uagcaccauuugaaauca............    23        0         seq
    ...................................................uagcaccauuugaaaucG............    13        1         seq
```

oar-miR-29b

```
cuucuggaagcugguuucacauggugg cuuagauuuuuccaucuuuguaucuagcaccauuugaaaucaguguuuuaggag
...................................................uagcaccauuugaaaucGg...........   61     1     seq
...................................................uagcaccauuugaaaucag...........   26     0     seq
...................................................uagcaccauuugaaaucagu..........   1977   0     seq
...................................................uagcaccauuugaaaucGgu..........   464    1     seq
...................................................uagcaccauuugaaaucagug.........   451    0     seq
...................................................uagcaccauuugaaaucagugu........   583    0     seq
...................................................uagcaccauuugaaaucaguguu.......   4534   0     seq
...................................................uagcaccauuugaaaucaguguuu......   7      0     seq
...................................................uagcaccauuugaaaucaguguuC......   3      1     seq
...................................................uagcaccauuugaaaucaguguuuu.....   2      0     seq
....................................................agcaccauuugaaaucag...........   1      0     seq
....................................................agcaccauuugaaaucagu..........   1      0     seq
....................................................agcaccauuugaaaucagugu........   1      0     seq
....................................................agcaccauuugaaaucaguguu.......   6      0     seq
.....................................................gcaccauuugaaaucagugu........   1      0     seq
.....................................................gcaccauuugaaaucaguguu.......   3      0     seq
........................................................ccauuugaaaucaguguu.......   1      0     seq
```
